# Nanoparticle Vaccines Against Infectious Diseases

**DOI:** 10.3389/fimmu.2018.02224

**Published:** 2018-10-04

**Authors:** Rashmirekha Pati, Maxim Shevtsov, Avinash Sonawane

**Affiliations:** ^1^School of Biotechnology, KIIT University, Bhubaneswar, India; ^2^Institute of Cytology of the Russian Academy of Sciences (RAS), St. Petersburg, Russia; ^3^Klinikum Rechts der Isar, Technical University of Munich, Munich, Germany; ^4^First Pavlov State Medical University of St.Petersburg, St. Petersburg, Russia; ^5^Discipline of Biosciences and Biomedical Engineering, Indian Institute of Technology Indore, Indore, India

**Keywords:** nanoparticles, vaccine development, human diseases, targeted vaccine delivery, antigens

## Abstract

Due to emergence of new variants of pathogenic micro-organisms the treatment and immunization of infectious diseases have become a great challenge in the past few years. In the context of vaccine development remarkable efforts have been made to develop new vaccines and also to improve the efficacy of existing vaccines against specific diseases. To date, some vaccines are developed from protein subunits or killed pathogens, whilst several vaccines are based on live-attenuated organisms, which carry the risk of regaining their pathogenicity under certain immunocompromised conditions. To avoid this, the development of risk-free effective vaccines in conjunction with adequate delivery systems are considered as an imperative need to obtain desired humoral and cell-mediated immunity against infectious diseases. In the last several years, the use of nanoparticle-based vaccines has received a great attention to improve vaccine efficacy, immunization strategies, and targeted delivery to achieve desired immune responses at the cellular level. To improve vaccine efficacy, these nanocarriers should protect the antigens from premature proteolytic degradation, facilitate antigen uptake and processing by antigen presenting cells, control release, and should be safe for human use. Nanocarriers composed of lipids, proteins, metals or polymers have already been used to attain some of these attributes. In this context, several physico-chemical properties of nanoparticles play an important role in the determination of vaccine efficacy. This review article focuses on the applications of nanocarrier-based vaccine formulations and the strategies used for the functionalization of nanoparticles to accomplish efficient delivery of vaccines in order to induce desired host immunity against infectious diseases.

## Introduction

**Figure 1:**
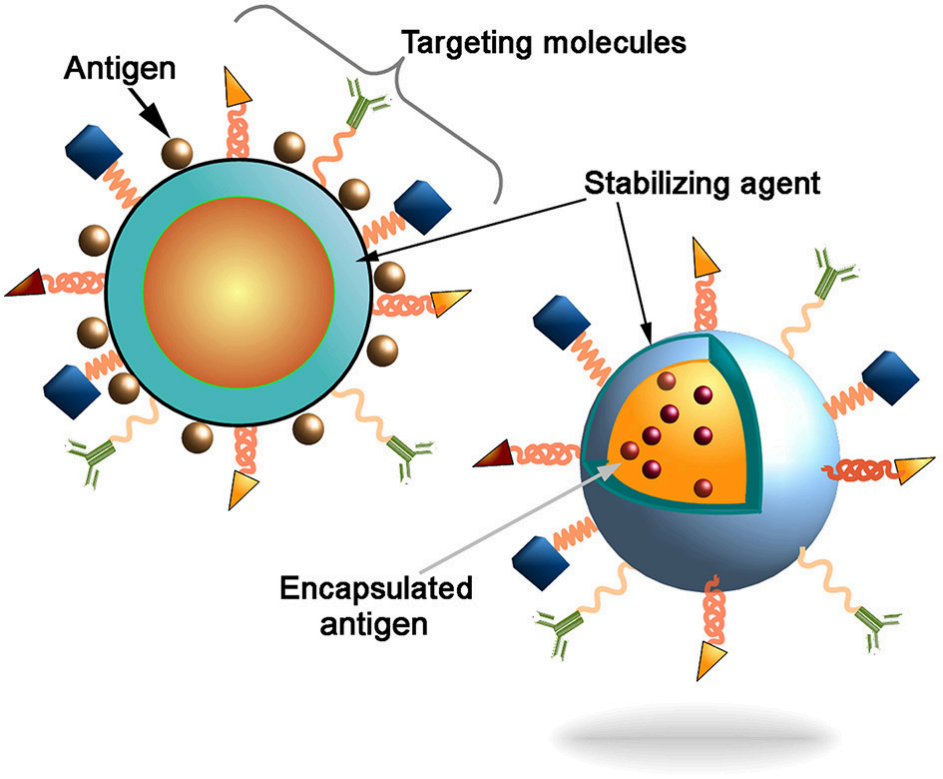
Schematic representation of the nanocarriers. Antigen can be conjugated to the nanoparticles surface or incapsulated into core of the particles. Decoration of the nanoparticles surface with targeting molecules (e.g., antibodies, Fab-fragments, peptides, etc) could further increase the delivery of particles into the antigen presenting cells (APCs) to induce innate and adaptive immune responses.

In twenty-first Century, infectious diseases have emerged as a serious threat to the health of millions of people across the globe (1). According to the World Health Organization (WHO) report for 2016, ~3.2 million deaths have occurred due to lower respiratory infections and 1.4 million from tuberculosis alone worldwide (2). Over the past few decades, many new infectious diseases have emerged and few old diseases re-emerged, which were once considered to be no longer a threat to the human being (3–5). Collectively, these diseases account for millions of deaths that cause enormous impact on the global socio-economical and health-care sectors. The major challenges to combat such diseases are that for many of them, there are no effective drugs available. One of the plausible approaches could be based on the application of nanocarrier based vaccination (6). However, there are still no effective vaccines available against some of the most prevalent diseases including immune deficiency syndrome (AIDS) and tuberculosis. This underlines an urgent need for the development of desired vaccines against these diseases. Some of the important aspects of any optimal vaccine includes (i) safety, (ii) stability, and (iii) the ability to elicit durable and adequate immune response with a minimum number of doses (7–9). Presently, different generation vaccines such as attenuated or killed whole organisms (*first generation*), subunit (*second generation*) and RNA or DNA vaccines (*third generation*) are used to elicit protective immunity against diseases (10–12). Despite several advantages of RNA or DNA vaccines such as minimal risk of infection, ability to elicit immune response against specific pathogen and cost effective (13); there are a number of challenges associated with the efficient delivery of these vaccine molecules to the target sites and the requirement of the prime-boost vaccination regimens with other immunogenic agents. These includes premature degradation of molecules and the inability to translate into a functional immunogen (14). Similarly, protein based vaccines are used successfully against several infectious diseases such as *Haemophilus influenza* type b, diphtheria, tetanus, acellular pertusis, meningococcus and pneumococcus (15), however they require an adjuvant to potentiate their immunogenicity, and also encounter early degradation after exposure to hostile milieu. Introduced recombinant protein-based vaccines (e.g., recombinant hemagglutinin vaccine for influenza) further enhance the immunity toward infection indicating the applicability of the recombinant technology for the vaccine production (16). To overcome these hurdles, an efficient vaccine delivery system is required which not only delivers the vaccine molecules to the target site to evoke enduring immune responses but also has minimal side effects and requires less doses. Moreover, there is an increasing need to develop new generation composite vaccine molecules that will act as immunogen as well as an adjuvant. Nanotechnology based formulations offer numerous advantages for the development of new generation vaccines. Nanocarrier based delivery system can protect the vaccines from premature degradation, improve stability, has good adjuvant properties, and also assists in targeted delivery of an immunogen to the antigen presenting cells (APCs). There are several mechanisms by which vaccines can be delivered to the specific sites using nanocarriers. Vaccine antigens can be encapsulated within the nanocarriers or decorated on their surface (Figure 1). Encapsulation within the nanoparticles (NPs) can protect the antigen from premature protease degradation and elicit sustainable release, whereas the surface adsorption facilitates their interaction with cognate surface receptors such as toll like receptors (TLRs) of APCs (17). Nanocarrier based delivery systems provide a suitable route of administration of vaccine molecules and enhance cellular uptake thereby resulting in robust innate, humoral, cellular as well as mucosal immune responses when compared with unconjugated antigens. This review mainly focuses on the potential use of nano delivery systems as novel vaccine strategies for the induction of innate as well as adaptive immune responses against infectious diseases.

## Key cellular components of the immune system

The immune system is composed of a collection of mobile cells that traffic throughout the body as well as reside at the site of entry (i.e., skin, respiratory, gastrointestinal, and genital tracts) in search of invading pathogens. These cells belong to two major types of innate and adaptive immune system. The innate immune cells like macrophages and neutrophils rapidly respond to the pathogens by recognizing pathogen surface moieties, phagocytosis, and the elimination of pathogens through activation of different antibacterial effector functions. Similarly, two major components of the adaptive immunity i.e., T and B-cells are important for the generation of cell mediated and humoral immune responses, respectively. T cells including CD4^+^ helper T cells secrete different cytokines to modulate the functions of B cells, whereas CD8^+^ T cells recognize and destroy virally infected cells. Antibodies produced by the B cells can further neutralize the invading microbes or clear infected cell or opsonized pathogens through cell-mediated systems. APCs, in particular dendritic cells (DCs) and macrophages, migrate through the body to sample, process and present the antigens to T-cells to activate cellular immune responses. These cells express various surface receptors to recognize cognate ligands and danger signals to trigger activation of different signaling pathways that eventually lead to the activation of T-cells (18). After sampling the antigens, DCs migrate from the peripheral tissues into the draining lymph nodes to activate naive T-cells (19), whereas macrophages after ingestion of antigens increase their lysosomal degradative machinery to enhance the antigen presentation to activate helper T cells.

## Types of nano-immuno activators

Some NPs are themselves able to stimulate different immune cells to boost the host immunity. The size, shape and surface chemistry of NPs (described below in more detail) are important factors that determine their potential to activate immune responses. In general, NPs are able to stimulate immune reactions by increasing the synthesis of defense genes and inflammatory reactions (20). Various types of NPs like gold, carbon, dendrimers, polymers and liposomes have the capability to induce cytokine and antibody responses (21–26). This was observed in the case of administration of empty PEGylated liposomes, which were able to elicit IgM response in an *in-vivo* model. (27, 28). Besides their potential to deliver various immune stimulators to the specific sites as well as into the deep tissues where vaccine molecules alone may not able to reach, these NPs have also been exploited as adjuvants to augment immunogenicity of vaccine candidates. Nano-immuno stimulators are the nano scale (20–100 nm) vaccine particles that can improve the vaccine efficacy *in vivo* better than bulk molecules (20, 29). Some of the known nano-immuno stimulators that have been used for this specific purpose are inorganic NPs (iron and silica) (30, 31), polymeric NPs (chitosan, PLGA, PVPONAlk, γ-PGA) (32–37), liposomes (cholesterol and lipids) (33, 38) and virus like particles (VLPs) (39, 40). Different types of NPs used to deliver antigens to give protection against different diseases have been listed in Table 1.

**Table 1:** List of antigens delivered by using different nanocarriers for the treatment of different diseases.

**Antigen**	**Nanocarrier used**	**Disease**	**References**
**AGAINST BACTERIAL INFECTION**			
Antigenic protein	Poly(D,L-lactic-co-glycolic acid) nanospheres	Anthrax	(41)
DNA encoding T cell epitopes of Esat-6 and FL	Chitosan Nanoparticle	Tuberculosis	(42)
Mycobacterium lipids	Chitosan Nanoparticle	Tuberculosis	(43)
Polysaccharides	Liposomes	Pneumonia	(44)
Bacterial toxic and parasitic protein	Liposomes	Cholera and Malaria	(45)
Fusion protein	Liposomes	*Helicobacter pylori* infection	(46)
Antigenic protein	Nanoemulsion	Cystic fibrosis	(47)
Antigenic protein	Nanoemulsion	Anthrax	(48)
Mycobacterium fusion protein	Liposome	Tuberculosis	(49)
**AGAINST VIRAL INFECTION**			
Antigenic protein	Chitosan Nanoparticles	Hepatitis B	(33)
Viral protein	Gold Nanoparticles	Foot and mouth disease	(50)
Membrane protein	Gold Nanoparticles	Influenza	(51)
Viral plasmid DNA	Gold Nanoparticles	HIV	(52)
Tetanus toxoid	Poly(D,L-lactic-co-glycolic acid) nanospheres	Tetanus	(53)
Hepatitis B surface antigen	Poly(D,L-lactic-co-glycolic acid) nanospheres	Hepatitis B	(54)
Hepatitis B surface antigen	Alginate coated chitosan Nanoparticle	Hepatitis B	(55)
Live virus vaccine	Chitosan Nanoparticles	Newcastle disease	(56)
Capsid protein	VLPs	Norwalk virus infection	(57)
Capsid protein	VLPs	Norwalk virus infection	(58)
Influenza virus structural protein	VLPs	Influenza	(59, 60, 61, 62, 63, 64)
Nucleocapsid protein	VLPs	Hepatitis	(65)
Fusion protein	VLPs	Human papilloma virus	(39, 40, 66, 67, 68)
Multiple proteins	VLPs	Rotavirus	(69, 70)
Virus proteins	VLPs	Blue tongue virus	(71)
Enveloped single protein	VLPs	HIV	(72, 73, 74, 75)
Viral protein	Polypeptide Nanoparticles	Corona virus for Severe acute respiratory syndrome (SARS)	(76)
**AGAINST PARASITIC INFECTION**			
Merozoite surface protein	Iron oxide Nanoparticles	Malaria	(30)
Epitope of *Plasmodium berghei* circumsporozoite protein.	Polypeptide Nanoparticles	Rhodent mamarial parasitic infection	(77)
Surface protein from *Eimeria falciformis* sporozoites	ISCOMs	Diarrhea	(78)

### Inorganic NPs

Some biocompatible inorganic NPs such as gold, carbon and silica have been exploited in the vaccine delivery studies (50, 79–81). These NPs can be synthesized in various shapes, size and surface modified forms. Some of the viral antigens were successfully delivered using inorganic NPs as carriers. This caused increase in antigen stability by protecting them from premature degradation by proteolytic enzymes. Delivery of viral and bacterial antigens using gold NPs was also found to induce quite robust host immune responses against influenza, immunodeficiency virus, foot and mouth, and tuberculosis diseases in mice (51, 52, 82, 83). Encapsulation of plasmid DNA that encode mycobacterial hsp65 antigen in gold NPs exhibited significant reduction in the *Mycobacterium tuberculosis*, causative agent of human tuberculosis, burden in infected mice (52, 82). Few studies have used hollow mesosporous silica, nanotube and spherical forms of carbon NPs as adjuvants to improve the immunogenicity and delivery of protein and peptide antigens against viral infections (79, 83, 84). Silica based NPs contain abundant silanol groups that can be utilized to introduce specific functional groups on their surface to gain access for vaccine molecules into target cells (84–86). The major advantages of inorganic NPs include low production cost, reproducibility and safety in application.

#### Polymeric NPs

In recent years, polymeric NPs have received great attention for their applications in the delivery of a number of vaccines. This is primarily due to their ease in preparation, biodegradability, biocompatibility, reduced cytotoxicity, and the possibility to fine-tune surface properties as needed (87). Moreover, it is relatively easy to control the rate of vaccine release by altering the composition or ratio of co-polymers during the NP synthesis process (87). The most commonly used polymeric NPs for vaccine delivery are poly (lactic-*co*-glycolic acid; PLGA) or poly (lactic acid; PLA). PLGA NPs have already been tried in the delivery of a broad range of antigens, including hydrophobic antigens (34, 35), hepatitis-B virus antigens (54), *Bacillus anthracis* (41), tetanus toxoid (35), and ovalbumin (88). The use of PLGA conjugated antigens exhibited strong immuno-stimulatory property by inducing cytokine and nitric oxide production against mycobacteria infection (89). In addition to synthetic polymers, some natural biopolymers such as alginate, pullans, inulins, and chitosan have been used as adjuvants (90–93). Inulin, a known activator of the complement cascade (94), conferred better protection against hepatitis B and influenza viruses (92, 93). Similarly, chitosan NPs were demonstrated as nanocarrier molecules for HBV antigens (55), DNA vaccine (56), and Newcastle disease vaccine (42). The delivery of PLGA and chitosan NP conjugated vaccine molecules enhanced the immune responses at the mucosal site (95, 96). Our recent study also showed that delivery of *M. tuberculosis* lipids using biocompatible chitosan NPs was able to induce significant humoral as well as cellular immune responses when compared to lipids alone in mice (43). We also found that intraperitoneal administration of these conjugates showed better activation of splenic T-cells. Another study by de Titta et al. has shown that intradermal administration of CpG conjugated polymeric NPs increased dendritic cell activation by several fold, exhibited comparable vaccine efficacy at ~400 times lower dose, and also caused enduring cellular immunity in comparison to free CpG (97). These desired properties along with already known reduced toxicity and biocompatibility under both *in vitro* and *in vivo* conditions make polymeric NPs plausible candidates for further preclinical pharmacokinetics and therapeutic applications (98).

#### Liposomes

In addition to polymeric NPs, liposomes are the second most widely explored vaccine and drug delivery vehicle in the nanomedicine field. The synthesis of liposomes is a spontaneous process, where hydration of lipids enables the lipid bilayer formation around an aqueous core (99). So far, different types of liposomes, including unilamellar or multilamellar vesicles composed of biodegradable phospholipids (e.g., phosphatidylserine, phosphatidylcholin and cholesterol) were included in the vaccine studies (100). Liposomes deliver vaccines by fusion with the target cell membrane (101).The structurally flexible and versatile liposomes are able to encapsulate both hydrophilic and hydrophobic substances. The hydrophilic molecules can be incorporated into the aqueous core, while hydrophobic molecules are encased within the phospholipid bilayer. Earlier reports have shown that delivery of antigenic proteins entrapped in multilamellar lipid vesicles elicit strong T and B-cell responses (102). Similarly antigenic peptides conjugated to phosphatidylserine (PS)-liposomes were readily internalized by APCs to potentiate T-helper cell mediated immune responses (103) and delivery of heat shock protein encoding vaccine DNA using liposomes elicited strong protective immunity against fungal infection (104). Because of their foreseen applications, several liposome based vaccine nano-formulations have been approved for clinical trials against intracellular pathogens, including viruses and *M. tuberculosis* (105). One such study already demonstrated the potency of liposomal aerosol carriers in the generation of protective immunity against *M. tuberculosis* infection (106, 107). Other studies have tried a combination of dimethyl dioctadecyl ammonium (DDA) lipid based liposomes and various immunomodulators to enhance immunity against influenza, chlamydia, erythrocytic-stage malaria, and tuberculosis infections (108–112). In the context of DNA vaccines, lipid-DNA complexes have been successfully delivered to the lungs of monkeys (101).

#### VLPs (virus like particles)

There are several reports that adequately proved applications of VLPs as a vaccine carrier, and also their ability to stimulate the host immune responses (113–115). VLPs are composed of self-assembled viral membrane that forms a monomeric complex displaying a high density of epitopes (115, 116). Interestingly, VLPs can also be engineered to express additional proteins either by fusion of proteins with the particles or by endogenous expression of multiple antigens (113, 117). It is also possible to chemically couple non-protein antigens and small organic molecules onto the viral surface to produce bioconjugates with VLPs (118, 119). Due to these distinct features, VLPs can provide protection not only against virus, but also against heterologous antigens (116). A specific immune response was successfully generated after the delivery of an antigen using virus capsid protein SV40 in mammalian cells (120). VLPs were also found to increase the immunogenicity of weak antigens. For example *Salmonella typhi* membrane antigen, influenza A M2 protein and H1V1 Nef gonadotropin releasing hormone (GnRH) assembled VLPs produced strong antigen specific humoral as well as cellular immune responses (121, 122). It is presumed that the use of VLP based nanoformulations could enable the antigens to achieve conformations resembling to native antigen structure, thus it may result in better stimulation of the host immune response (122).

#### Dendrimers

Dendrimers are three dimensional, mono-dispersed and hyperbranched nano structures that are made up of a mixture of amines and amides. Few studies have explored the application of dendrimers in the delivery of different antigenic molecules. The most commonly used dendrimers for vaccine delivery are polypropyleneimine (PPI) and polyamido amine (PAMAM) dendrimers. A single dose of dendrimer encapsulated multiple antigens was found to produce strong antibody and T-cell responses against Ebola virus, H1N1 influenza, and *Toxoplasma gondii* (123). This generation of robust immune response was found to be due to efficient uptake of dendrimers by the host cells. Similarly a significant increase in the vaccine efficacy of HIV transactivator of transcription (TAT) based DNA vaccine was observed due to enhanced cellular uptake of PMAM dendrimer (124). Hence, the possibility to tailor the dendrimers to attain certain biological and physico-chemical properties, and also the feasibility to conjugate several ligands to the single molecule have made dendrimers promising candidates for the development of new generation vaccines with enhanced immunogenic properties.

## Delivery of immune stimulators using nanocarriers

### Cytokines

Cytokines are known as important signaling molecules secreted by different cells in response to external stimuli. Some of the cytokines are able to activate immune cells to generate protective immunity against several diseases. However, cytokines are mostly susceptible to early degradation that subdue their participation in the generation of host immunity. Moreover, uncontrolled release of cytokines as immune responders may sometimes lead to harmful side effects (125). To overcome these limitations, several studies have attempted to synthesize engineered nanocarriers to achieve effective and controlled delivery of cytokines to the target sites. This approach was found to reduce their toxicity, improve circulation time and antigen specific T-cell responses in comparison to free cytokines (126, 127). Incorporation of granulocyte macrophage colony stimulating factor (GM-CSF) and interferon alpha (IFN-α) into nano-carriers exhibited great application in cancer therapy (128, 129). Nano-carrier conjugated cytokines also showed great potential in the treatment of infectious diseases. For example, IL-12 encapsulated microspheres induced strong protective immunity against tuberculosis (130). This effect was due to production of high antibody titers as a result of sustained and controlled release of IL-12 from the microspheres in immunized mice (130).

### Toll like receptor agonists

Like cytokines, several toll-like receptor (TLR) agonists were also explored as immune activators to augment immune surveillance mechanisms. Different immune effector cells such as macrophages, B-cells and DCs express different types of TLRs, which are known to interact with specific pathogen associated molecular patterns (PAMPs). These specific interactions eventually initiate downstream signaling cascades to ensure the elimination or generation of immunity against pathogens (131, 132). Conjugation of TLR specific agonists on nanocarriers helps to target the molecules to specific immune cells and therefore reduce the possibility of systemic biodistribution. One such study has shown that conjugation of TLR-7/8 agonist on nano polymers caused efficient internalization by APCs and also prolonged the T cell responses (133). Administration of NPs loaded with vaccine peptide antigen and TLR-7 and 9 ligands were also found to induce strong memory and effector CD8^+^ T-cell response (134). Another study has shown that conjugation of TLR-8 agonist to a polymer nanocarrier increased activation and maturation of naive DCs due to selective endocytosis and prolonged release of an immunogen by the nanocarrier inside DCs (135). Moreover, intradermal injection of CpG and antigen encapsulated polymeric NPs were rapidly drained into the lymph nodes to activate DCs (97). These studies indicate that NPs can be used as a tool to appropriately target presentation of antigens to T and B-cell rich lymphoid organs.

### Nucleic acids

The genetic molecules such as DNA, plasmids and RNA can also act as immuno-stimulants. Due to these characteristics, in addition to less risk to cause disease particularly in immunocompromised individuals, these genetic materials are considered as promising candidates for the development of next generation vaccines. After administration, the plasmid vector translocates to the nucleus to initiate transcription of recombinant genes using the host cellular machinery. A recombinant DNA segment encoding HspX-PPE44-esxV fusion antigen of *M. tuberculosis* showed great potential as a new tuberculosis DNA vaccine candidate (136). A similar type of study has been conducted in the past where the vaccination of DNA or RNA constructs expressing mycobacterium antigens were capable of inducing humoral as well as cellular immune responses (137). Likewise, plasmids harboring genes encoding for viral antigen have been encapsulated into alginate nanocarriers and targeted against viral infections (138).

## Importance of physicochemical properties in designing nano-immuno formulations

In order to improve their delivery and vaccine characteristics, different approaches have been practiced to conjugate vaccine molecules to different nanocarriers. Vaccine molecules can be surface conjugated, encapsulated or surface adsorbed with the nanocarriers. Antigen adsorption on the nanocarrier is simply based on the presence of a charge or hydrophobic interactions between NPs and the candidate molecule (139, 140). This type of interaction is usually non-covalent, which may lead to rapid dissociation of antigens from nanocarriers depending upon the external milieu such as pH, ionic strength, temperature, and the antigen hydrophobicity. On the other hand, encapsulation and chemical conjugation of antigen to nanocarriers is more stable due to strong interactions and chemical bond formation between the target molecule and the nanocarrier. Further, antigens can also be encapsulated into nanocarriers by simple mixing reaction during the synthesis. In this case, the antigens are released only after partial or complete dissociation of the nanocarrier (141). These processes have already been used with silica and gold NPs (142). Similarly, chitosan and dextran sulfate NPs were used for the preparation of cationic and anionic antigenic formulations. Some viral antigens are known to bind to both positive as well as negative charged NPs through immobilization process and hydrogen bonds (143). The immobilization process depends on the charge, pH, ratio of NPs and antigens, and the protein partition coefficient between the solution and the colloid (143). Several antigens were successfully delivered to the target sites by chemical conjugation, adsorption and encapsulation to soft nanocarriers like VLPs, liposomes and immune stimulating complexes (ISCOM) (144–147). ISCOMs are a class of adjuvant formulations that consist of saponins, cholesterol and phospholipids in specific ratios. Antigens can be formulated into ISCOMs directly (148) or after the surface modification (149, 150). Since ISCOM particles are negatively charged, direct conjugation of most of the soluble proteins is a limiting factor. Nanocarriers can augment immunogenicity of a molecule. For example, influenza antigen H1N1 conjugated chitosan NPs and *Yersinia pestis* F1-antigen coated gold NPs (AuNPs) produced higher levels of antibody and cytokine responses in comparison to mice administered with unconjugated antigens (151).This was found to be due to stabilization and increased immunogenicity of vaccine antigens due to conjugation with NPs.

Another important aspect in the development of nano-immuno formulations is that they improve antigen delivery and presentation (152). In this context, NP shape, size and surface charge are key factors that affect NP circulation, biodistribution, bioavailability and specificity by crossing biological barriers. Besides these factors, particle geometry such as surface to volume ratio plays an important role in the determination of immunogen release and degradation kinetics (153, 154). Here, the importance of different physicochemical parameters such as size, shape, surface area, porosity, hydrophobicity, hydrophilicity and crystallinity in the interaction between NPs and the target cell is discussed.

### Size

The size of NPs determines the mode of cellular uptake and specificity (155, 156). PLGA NPs of large size (1, 7 and 17 μm) showed reduced internalization rate in comparison to smaller NPs (300 nm) (157). The size of NPs also determines the cellular specificity and migration. Smaller NPs (20–200 nm) were readily endocytosed by the resident DCs, whereas larger size (500–2,000 nm) NPs were effectively taken up by the migratory DCs (158). NPs of less than 200 nm size were drained into the lymph nodes (159), while particles up to 20 nm range were suitably transported to the APCs (152, 160). Notably, NP curvature also affects the cellular interaction and phagocytosis rate (161). NPs of 150 nm diameter and 450 nm height showed more cellular uptake as compared to the particles having 1,200 × 200 nm size. Of note the size of NPs was also found to influence the activation of signaling pathways. A study has demonstrated that smaller NPs are able to alter the cell signaling processes more efficaciously than the large NPs (31).

### Surface charge

Vaccine loaded NPs can also be targeted to specific sites by modifying the NP surface charge. Delivery of such NPs at appropriate sites elicit strong immune responses against antigens. NP surface charge is responsible for the interaction with congnate surface molecules present on the target cells. This was exemplified from the observation that cationic polysterene NPs were efficiently internalized by the APCs in comparison to neutral surface charged NPs. This may be due to electrostatic interactions between the cationic NPs with anionic cell membranes (162, 163). Interestingly, pulmonary instillation of cationic and anionic NPs showed similar endocytosis rate in macrophages and draining lymph nodes, however cationic formulations showed more expression of *Ccl2* and *Cxc10* chemokines that caused more recruitment and maturation of CD11b DCs in comparison to anionic NPs in the lung (125, 156). Similarly, neutral silica-silane shell polymer NPs were less effective in the activation of innate immune cells (128). These studies clearly indicate appropriate surface modifications of NPs may help to generate stronger immunological responses against specific infection.

### Shape

Beside size and surface charge, NP shape is also a critical determinant in the cellular interaction, intracellular trafficking and the rate of antigen release inside the host cells (79, 141). Spherical gold NPs were actively internalized by bone marrow derived dendritic cells in comparison to rod shaped particles of similar dimensions (33, 34), and that spherical NPs were able to induce strong immune response than cube or rod shaped NPs (164). Another study reported that worm-like particles were impaired in phagocytosis as compared to spherical NPs (151). These distinctions were ascribed to the differences in contact area between NPs and the target cell membrane. The shape of NPs also determines the localization of NPs inside the host cells. This was demonstrated by the fact that although nano rods and nano sheets were internalized via clathrin mediated endocytosis, nano rods were particularly delivered to the nucleus while nano-sheet were retained in the cytoplasm (146, 147, 155). This is an important aspect in the context of improving antigen processing and presentation to T-cells. It is well established that enhanced antigen processing and presentation can be achieved if the candidate molecules are delivered to the lysosomal compartment of the cells.

### Hydrophobicity

Hydrophobicity of NPs plays a significant role in the interaction with soluble proteins and immune cells through recognition of hydrophobic moieties (165). Previous studies have shown that hydrophobic polymeric NPs are strong inducers of cytokines and co-stimulatory molecules than hydrophilic polymeric NPs (53, 105, 166). Exposure to hydrophobic NPs showed enhanced activation of DCs by inducing the expression of CD86 co-stimulatory molecules when compared with hydrophilic ones. Similar observations were reported in other innate immune cells, in which hydrophobic NPs were able to activate these cells by up-regulating the expression of proinflammatory cytokine encoding genes (102), and also facilitated opsonization process by increasing the adsorption of immunoglobulins on the cell surfaces (103). However, other studies have reported that polyethylene glycol coating (PEGylation) reduced the interaction of NPs with immune receptors (50, 80). This property is considered useful in the prevention of non-specific adsorption of proteins on NPs and thereby prevent their up-take by APCs (50). Such non-specific adsorption of proteins and their uptake by phagocytic cells can also be preventing by the incorporation of an alkyl linker between the PEG and thiol moieties on NPs (80).

### Surface modification

Surface modification of NPs alters ligand specificity and interaction with APCs (160). Conjugation of CD47 molecules on the surface of NPs modulated the down-stream signaling cascades and also reduced NP internalization by phagocytic cells (131). Functionalization of NPs with TLR-7, TLR-8, and TLR-9 agonists increased cytokine production and the expression of immunoregulatory genes (132–134). Similarly, conjugation of poly (methyl vinyl ether-co-maleic anhydride; PVMA), TLR2, and TLR4 agonists, and galactose polymer to NPs were shown to activate the complement pathway as a result of stable binding to C3b complement factor (139, 142). Further, lipoprotein-like NPs showed LPS scavenging activity, thereby resulting in the inhibition of TLR-4 dependent inflammatory responses (140). Overall, these studies strongly demonstrated that tuning of physico-chemical properties of NPs could be used as a fundamental tool to target vaccine molecules to specific sites to induce desired immune responses.

## Implications of the nanocarriers in the vaccine development

**Figure 2:**
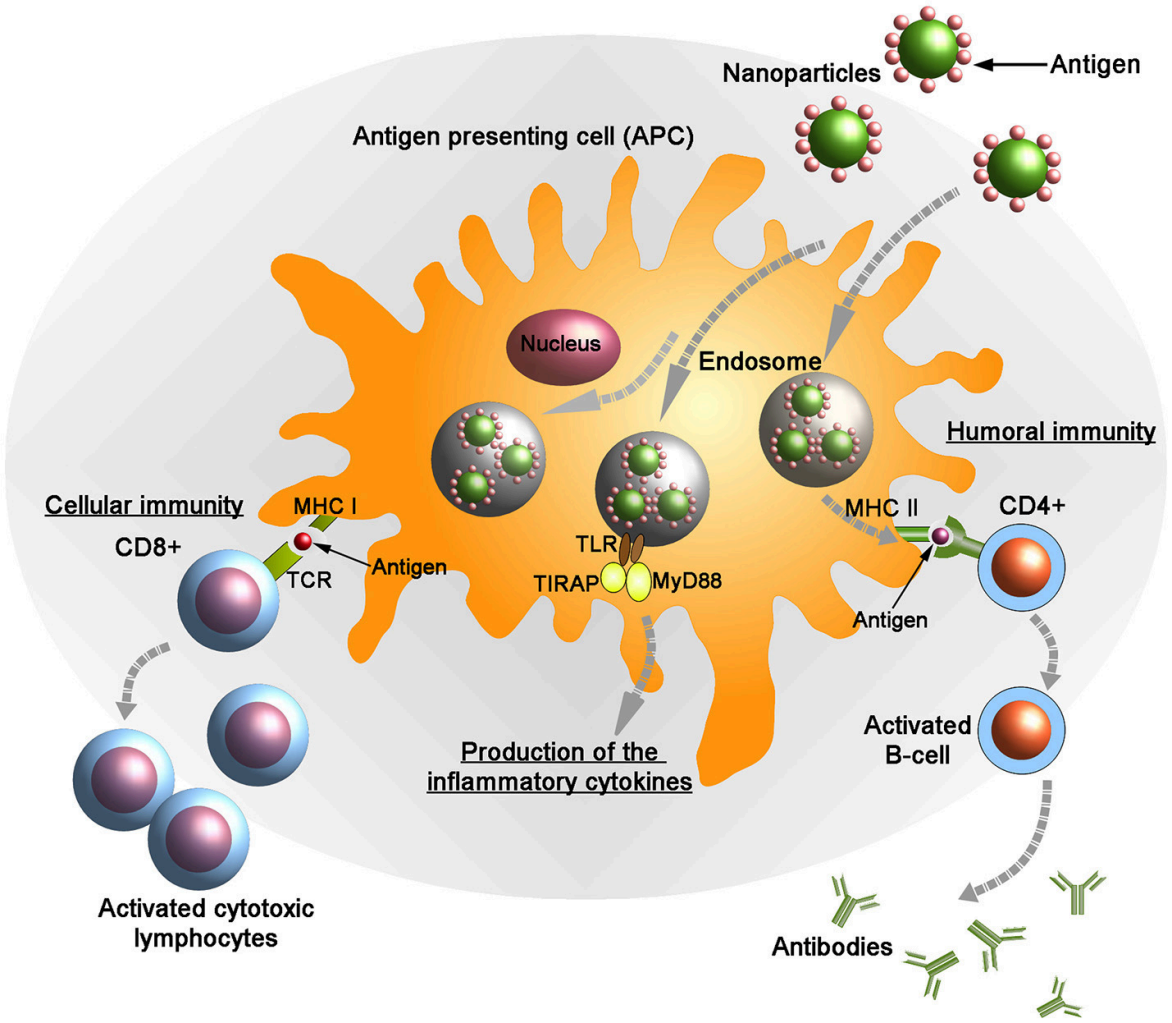
Targeted delivery of antigenic molecules using surface engineered nanoparticles into the antigen presenting cells (APCs). Endogenously generated antigens are presented in complex with class I major histocompatibility complex (MHC I) on the membrane of APCs to CD8+ T lymphocytes. Following the interaction between MHC I and T-cell receptor (TCR) in presence of co-stimulatory molecules and cytokines the activated CD8+ cells kill the infected cells by inducing cytotoxicity. Also the antigens are presented on the APC surface by class II MHC molecules to the helper (CD4+) T cells. Subsequently, CD4+ cells activate B-cells that produce anti-microbial antibodies. Upon stimulation the adaptor proteins MyD88 (myeloid differentiation marker 88) and TIRAP (TIR domain containing adaptor protein) colocalize with TLR (toll-like receptor) allowing for activation of the NF-κB pathway and leading to the production of pro-inflammatory cytokines.

Emerging studies have proved that nanocarriers can be useful mediators in the development of vaccines against various diseases. In this context, it is important to develop NP formulations that can deliver immunogens to APCs especially DCs to induce effective antigen-specific T-cell responses (Figure 2). Several nanocarriers have been shown to specifically activate DCs to effectuate anti-tumor or anti-viral immune responses (167–170). Zhu et al. proposed that nano-TiO_2_ and Fe_3_O_4_-TiO_2_ particles could function as a useful vector to promote vaccine delivery in immune cells (168). Co-incubation of nano-TiO_2_ and Fe_3_O_4_-TiO_2_ with DCs resulted in an increased production of TNF-α, and also upregulated the expression of CD80, CD86 and MHC class II molecules through the NF-κB signaling pathway (163). In this way, immunization efficacy of various NP formulations such as erythrocyte membrane-enveloped poly(D,L-lactide-co-glycolide) (PLGA) NPs for antigenic peptide (hgp10025-33) and TLR-4 agonist, VLPs expressing RSV glycoproteins, chitosan-coated EphrinA1-PE38/GM-CSF, and several others have been improved (171–177). NPs can also control cell polarization and differentiation. Branched polyethylenimine-superparamagnetic iron oxide NPs (bPEI-SPIONs) promoted Th1 polarization of DCs (178). Another study by Sehgal et al. showed that NPs can also be used to target subsets of particular immune cells. They have shown that simultaneous targeting of DC subsets (i.e., DC-SIGN+ and BDCA3+DC) by NPs synergistically stimulated the activation of T cell-mediated immunity when compared with targeting of each DC subset separately (170).

Preclinical studies by different research groups have successfully demonstrated the efficacy of NP based vaccines in the induction of specific immune responses against tuberculosis (42, 179–182). Feng et al. developed a NP-based recombinant DNA vaccine that consists of Esat-6 and fms-like tyrosine kinase 3 ligand enveloped with chitosan NPs (42). Intramuscular prime vaccination followed by nasal boost of this recombinant DNA vaccine remarkably enhanced T cell responses in *Mycobacterium tuberculosis* challenged mice (42). Another study has shown that pulmonary administration of *M. tuberculosis* Ag85B antigen and CpG adjuvant conjugated polypropylene sulfide NPs (NP-Ag85B) induced *M. tuberculosis* specific polyfunctional Th1 responses and also reduced the lung bacterial burden (183).

## Targeted delivery of nanoparticles can activate innate and adaptive immune responses

### Innate immunity

Macrophages and monocytes are highly heterologous cells that are distributed throughout the body. Macrophages process and present the antigens to elicit adaptive immune response. Due to their intrinsic phagocytic nature, macrophages can be easily targeted by surface engineered NPs, in which cognate ligands agonist to macrophage receptors can be conjugated on the NP surface (Figure 1). As discussed above several physico-chemical parameters of NPs such as size, surface charge, hydrophobicity, surface topography, and material composition can be optimized to facilitate the interactions between NPs and macrophage receptors (184–186). The rate of NP endocytosis also depends upon the type of cell surface receptors and the ligand conjugated to the NP surface. For example, NPs targeted via mannose and Fc receptors were rapidly internalized as compared to scavenger receptors (187). Endocytosis of IgG and anti-F4/80 antibody coated NPs showed more uptake rate and retention time inside the macrophages without affecting the cell viability (188, 189). Also, positively charged NPs interact more strongly with negatively charged phospholipid components of the cell membrane (190). Hyperactivation of some inflammatory cells can also be restricted through controlled release of stimulants using NPs. Upon activation, neutrophils can secrete variety of cytokines and hydrolytic enzymes in response to infection (191). Prolonged neutrophil activation often leads to acute inflammation and tissue damage at the localized site. Therefore, controlled release of molecules is necessary to prevent the hyperactivation and massive recruitment of neutrophils. It has been reported that bovine serum albumin (BSA) NPs were able to modulate the functions of neutrophils following their internalization. Intravenous injection of anti-inflammatory peptide encapsulated polymeric NPs reduced neutrophil recruitment and subsequently hyperinflammation to prevent further tissue damage (192). The use of NPs to deliver vaccine/drugs in a controlled fashion is now considered as an attractive approach to develop therapeutic strategies against a range of acute and chronic inflammatory diseases (193).

### Adpative immunity

T and B-cells of the adaptive immune system express a repertoire of receptors to recognize a range of antigens. Activation or suppression of T-cell immunity can determine the fate of a disease. A number of NP based therapeutic strategies have been developed to regulate T-cell activity against viral, bacterial, or fungal infections. For example, antiviral siRNA or retroviral drug encapsulated lipid NPs or dendrimers were effectively delivered to CD4^+^ T-cells to block HIV replication. This caused a significant reduction in HIV titer when compared with the use of non-encapsulated retroviral drugs (191, 194). T-cell activation also depends up on the type and size of NP used for the delivery of antigen. Liposome encapsulated antigens were better presented to CD4^+^ T cells by APCs (195, 196) and delivery of 200 nm ova conjugated NPs increased MHC class I and II expression and also produced a higher percentage of antigen specific CD4^+^T cells as compared to 30 nm ova conjugated particles (197).

B cells are able to recognize and respond to the microbial surface antigens through B-cell receptors (198). Activation and clonal expansion of antigen specific B-cells using engineered NPs have been exploited for the development of vaccines against different diseases (Figure 2). Encapsulation of antigen in virus like particles (VLPs) was able to induce strong and durable humoral responses when compared with the administration of exposed vaccine molecules (199). The potency of immune responses also depends upon the mode of antigen presentation to the target cells. Surface conjugated immunogenic proteins and peptides were able to activate B cells much stronger than encapsulated antigens (200). A single dose of PLGA NPs with surface displayed ovalbumin (OVA) elicited strong antibody responses *in vivo* as compared to free OVA (201, 202). NPs can also be used to activate specific immune responses. A study has shown that peptide conjugated carbon nanotubes showed significant antigen specific IgG response in comparison to peptide or adjuvant alone (83).

## Nanoparticles can be used to increase cross antigen presentation

In general, antigens captured by APCs from the extracellular environment are targeted to the endo-lysosomal compartments, where they are first processed into peptides and then loaded onto class II MHC molecules before presentation to CD4^+^ helper T cells. However, cytosolic antigens are loaded on MHC class I molecules and presented to CD8^+^ T-cells, which are crucial for the clearance of viral and intracellular infections (203). It is reported that some fraction of antigens delivered through NPs are trafficked to cytosolic vacuoles of APCs and presented by MHC class I molecules (203–205). The NP mediated cross antigen presentation was first demonstrated in antigens conjugated to iron oxide polymer NPs (206–209). In addition, inorganic and polymeric NPs have also been used for antigen delivery to cytosol (210,–212). In this context, lipid NPs were shown to induce CD8^+^ T cell expansion by efficient antigen cross presentation against viral infection in *in-vivo* models (102, 213). Similarly, invariant natural killer T cells (iNKT), which are a special subset of T-cells, recognize lipid antigens presented by CD1d cells. PLGA NPs conjugated with α-galactosylceramide glycolipid, an iNKT cell stimulant, increased cytokine release as well as expansion of antigen specific CD8+ T cells (214). The cross antigen presentation also depends upon the particle-antigen linkages. It has been shown that disulfide bonding between NP and antigens caused release of antigens into the endosomal compartment and also enhanced CD8^+^ T cell formation as compared to non-degradable linkers (215, 216). Similarly, pulmonary administration of NPs efficiently enhanced cross antigen presentation, which resulted in at least 10-fold more effector CD8^+^T cells in lungs (217).

## Nanoparticles as adjuvants to generate immune responses in lymphoid organs

Adjuvants are known to enhance and prolong the immune responses against antigens. Delivery of adjuvants and antigens using NPs have been found useful to prolong their exposure in the lymphoid organs such as lymph nodes to generate robust immune responses. This is especially important for small adjuvant molecules, which are rapidly cleared from the bloodstream. NPs with a size ranging from 10–100 nm can penetrate the extracellular matrix and travel to the lymph nodes where they can be internalized by the resident macrophages to activate T-cell responses (218–220). The bio-distribution of NPs also depends upon the route of administration and size. It was observed that larger particles accumulated near the site of NPs and were subsequently endocytosed by the local APCs (160), whereas the smaller NPs drained to the blood capillaries (158, 218). PEG coated liposomes of 80–90 nm diameter showed higher accumulation in lymph nodes after subcutaneous administration as compared to intravenous and intraperitoneal administration (221).

## Conclusions

The nano-immuno formulations can improve the antigen stability, targeted delivery and also enhance their immunogenicity properties. Most soluble antigens cannot be efficiently endocytosed by the APCs and hence are poorly effective in inducing protective immunity. The immunogenicity of such soluble vaccine antigens can be improved by conjugating them with nanocarriers that can facilitate the recognition and uptake by APCs. This strategy has already been proved effective for inducing/increasing the immunogenicity of poorly immunogenic antigens, such as polysaccharides of pneumococcal vaccines (222). In the last few years, the application of nanotechnology in the field of immune engineering is growing rapidly with a number of new carrier synthesis strategies. Furthermore, novel nano formulations also contain immunostimulatory molecules to enhance the adjuvant properties of the nanoparticles. Co-encapsulation of the TLR agonists [e.g., CpG, poly(I:C)] (77) or imiquimoid (78) into dextran or chitosan NPs, respectively enhanced receptor-based recognition of the nanovaccines with subsequent cell activation. The recent study by Margaroni et al. showed that vaccination with poly(D,L-lactide-co-glycolide; PLGA) nanoparticles with *Leishmania infantum* antigens (sLiAg) and surface-modified with a TNFα-mimicking eight-amino-acid peptide (p8) induced significant protection against parasite infection in BALB/c mice accompanied by activation of CD8+ T cells and increase in IFNγ production (223).

Additionally, NPs can be tailored for non-invasive administration and prolonged delivery of the vaccine antigens to a specific location, thus providing the possibility for formulation of the single dose vaccine. Several studies clearly demonstrated the efficacy of the non-invasively administered vaccines such as intranasal application of influenza nano vaccine (224), chitosan NPs with hemagglutinin protein of H1N1 influenza virus (225), *Streptococcus equi* proteins (226), hepatitis B surface antigen (pRc/CMV-HBs) (227) and plasmid encoding a multi-epitope protein against *M. tuberculosis* (pHSP65pep) (228) or antigen 85B (229) were used to provide protective immunity against infections. These considerations can improve the progress of ongoing strategies in the development of nanoparticle-based vaccines. In future, development of nanovaccines will address not only the possibility to induce the immune response but also the anti-infective therapeutic activity of NPs thus representing the feasibility to apply multifunctional particles for the treatment of diseases.

## Author contributions

AS and RP wrote the manuscript. AS supervised the process. MS wrote the part on Use of nanocarriers in vaccine delivery to dendritic cells.

### Conflict of interest statement

The authors declare that the research was conducted in the absence of any commercial or financial relationships that could be construed as a potential conflict of interest.
